# Identification and characterization of long non-coding RNA (lncRNA) in the developing seeds of *Jatropha curcas*

**DOI:** 10.1038/s41598-020-67410-x

**Published:** 2020-06-25

**Authors:** Xihuan Yan, Lanqing Ma, MingFeng Yang

**Affiliations:** 1 Beijing Advanced Innovation Center for Tree Breeding by Molecular Design, Beijing University of Agriculture, Beijing, 102206 People’s Republic of China; 2 Key Laboratory for Northern Urban, Agriculture of Ministry of Agriculture and Rural Affairs, Beijing University of Agriculture, Beijing, 102206 People’s Republic of China

**Keywords:** Plant embryogenesis, Plant molecular biology, Plant physiology

## Abstract

Long non-coding RNAs (lncRNAs) play critical roles in plant development. However, the information of lncRNAs in *Jatropha curcas* remains largely unexplored. Thus, an attempt has been made in *J. curcas* to identify 1,850 lncRNAs based on deep sequencing of developing seeds at three typical stages. About ten percent lncRNAs (196 lncRNAs) were differentially expressed lncRNAs during seed developing process. Together with reverse transcription quantitative real-time PCR, the lncRNA expression analyses revealed the stage-specific expression patterns of some novel lncRNAs in *J. curcas*. The target genes of lncRNAs were annotated for their roles in various biological processes such as gene expression, metabolism, and cell growth. Besides, 10 lncRNAs were identified as the precursors of microRNAs and 26 lncRNAs were predicted to be the targets of *Jatropha* miRNAs. A total of 31 key lncRNAs play critical roles in the seed developing process in the context of cell growth and development, lipid metabolism, and seed maturation. Our study provides the first systematic study of lncRNAs in the developing seeds of *J. curcas* and facilitates the functional research of plant lncRNAs and the regulation of seed development.

## Introduction

*Jatropha curcas* is a perennial tree belonging to Euphorbiaceae family and its seed has a high content of oil which can be used as biodiesel^1,2^. Gene expression profiles have been analyzed by several efforts in the developing seeds of *Jatropha* in order to understand the molecular processes of oil metabolism and seed development^3–6^. The whole genome sequence of *J. curcas* have been sequenced and assembled independently by Japan, China and South Korea, and these efforts lay a solid foundation for further exploration of the non-coding RNAs of *J. curcas*^7–9^.

Non-coding RNAs, including microRNAs (miRNAs) and long non-coding RNAs (lncRNAs), are functional transcripts that are not translated into proteins but usually function to regulate the expression of other genes. Studies demonstrate that lncRNAs regulate gene expression in plants by DNA methylation and chromatin remodeling, and in some cases, they act as miRNA sponges to enhance the expression of mRNA targeted by miRNA^10^. A great body of evidence indicates that lncRNAs function in both nuclear and cytoplasmic compartments and play essential regulatory roles in plant development processes (the development of pollen, fiber and lateral root; photomorphogenesis), plant reproduction processes (vernalization, flowering time and male sterility), and plant stress responses^11–15^. The role of lncRNAs in plant seed development has been acknowleged in recent years, as much effort has been made to identify the lncRNAs from seeds in many plants, including maize^16–18^, *Brassica napus*^19^, tree peony^20^, castor bean^21^, pigeonpea^22^, *Ginkgo biloba*^23^, and rice^24^. In maize, lncRNAs might play a part in the complex regulation of genetic imprinting during maize endosperm development^17^, and lncRNAs probably have function in lipid metabolism regulation of *Brassica napus* and tree peony developing seeds^19,20^. Previous work also indicates that lncRNAs impact on developmental and metabolic processes as endogenous target mimics which leads to sequestering of the miRNAs^18,22^. In all, these reports support the fact that lncRNAs play critical roles in regulating seed development including endosperm, embryo, and fruits.

Although much effort has recently been made to identify the function of protein-coding genes and miRNAs in *J. curcas*^25–27^, the identification and role of lncRNAs have not been revealed in this biodiesel tree. Furthermore, lncRNAs are not conserved between plant species, and the potential functions of lncRNAs in plant seeds remain largely unclear, especially in endosperm of oil seeds. Therefore, it is necessary to discover novel lncRNAs and analyze their function in the developing seeds of *J. curcas*.

The present study aims to identify lncRNAs and compare their differential expression in the developing seeds of Jatropha. Deep sequencing was carried out in the seeds at three typical developmental stages, and functional annotation was then performed on the targets of differentially expressed lncRNAs to examine their possible roles in seed developing process.

## Results and discussion

### Quality assessment of sequence data and transcripts assembly

To investigate lncRNA and their expression profiles in the developing seeds of *J. curcas*, we sequenced nine lncRNA libraries from three stages of seed development (small, middle and large seeds representing young, intermediate and mature respectively) with three biological replicates. After trimming the adaptors, filtering out poly-N regions and low-quality reads, the reads quality was checked based on Q-score, and the percentage of Q30 base was 96.53% or more (Table 1). A total of 107.39 Gb clean reads was obtained from nine samples with at least 10.28 Gb for each sample. After mapping the clean reads to the *Jatropha* genome JatCur_1.0 (https://www.ncbi.nlm.nih.gov/assembly/GCF_000696525.1/), the transcripts including protein-coding RNAs and non-coding RNAs were assembled.

**Table 1:** Transcriptome sequencing results of *J. curcas* developing seeds.

Samples	Read sum	Base sum	GC (%)	N (%)	Q30 (%)
L1	41,926,133	10,467,410,236	45.94	0.02	97.09
L2	48,461,287	12,097,611,214	45.84	0.02	97.16
L3	55,708,854	13,907,728,138	45.41	0.02	96.68
M1	48,304,848	12,058,792,600	44.2	0.02	96.53
M2	47,086,619	11,756,937,760	44.64	0.02	96.53
M3	41,154,388	10,275,769,312	44.21	0.02	96.84
S1	53,487,415	13,353,591,886	44.44	0.02	96.89
S2	45,298,184	11,312,135,322	45.07	0.02	96.89
S3	48,634,140	12,145,143,996	45.58	0.02	96.83

L1, L2 and L3 are larger seeds; M1, M2 and M3 are middle seeds; S1, S2 and S3 are small seeds; Read Sum: the statistical count of pair-end reads in clean data. Base Sum: total bases in clean data; GC (%): GC content in clean data; N (%): the percentage of unidentified bases in clean data; Q30 corresponds to a base calling accuracy of 99.9%.

### Identification of *J. curcas* lncRNAs and the characteristic features

The prediction of new lncRNA includes two steps: basic screening and potential coding ability screening. Transcripts lengths, exon numbers, and FPKM were considered in the step of basic screening. Transcripts with FPKM more than 0.5 are usually considered to be convincing expression in RNA sequencing studies. In this report, transcripts with lengths > 200 nt, exons ≥ 2, and FPKM ≥ 0.5 were selected as lncRNA candidates. By basic screening, the sequence information of lncRNA candidates was ready for potential coding ability screening.

**Figure 1:**
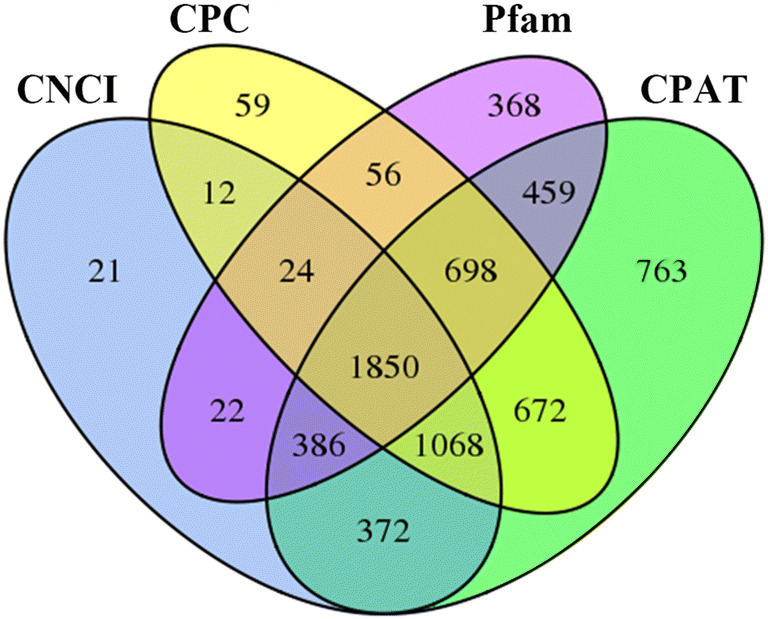
The Venn diagram of the noncoding transcripts identified by CPC, CNCI, CPAT, and Pfam analyses. Each circle represents an analysis method for predicting lncRNA. The number in the circle represents the number of transcripts predicted to be lncRNAs. The intersection of four circles is taken as the final result of lncRNAs.

**Figure 2:**
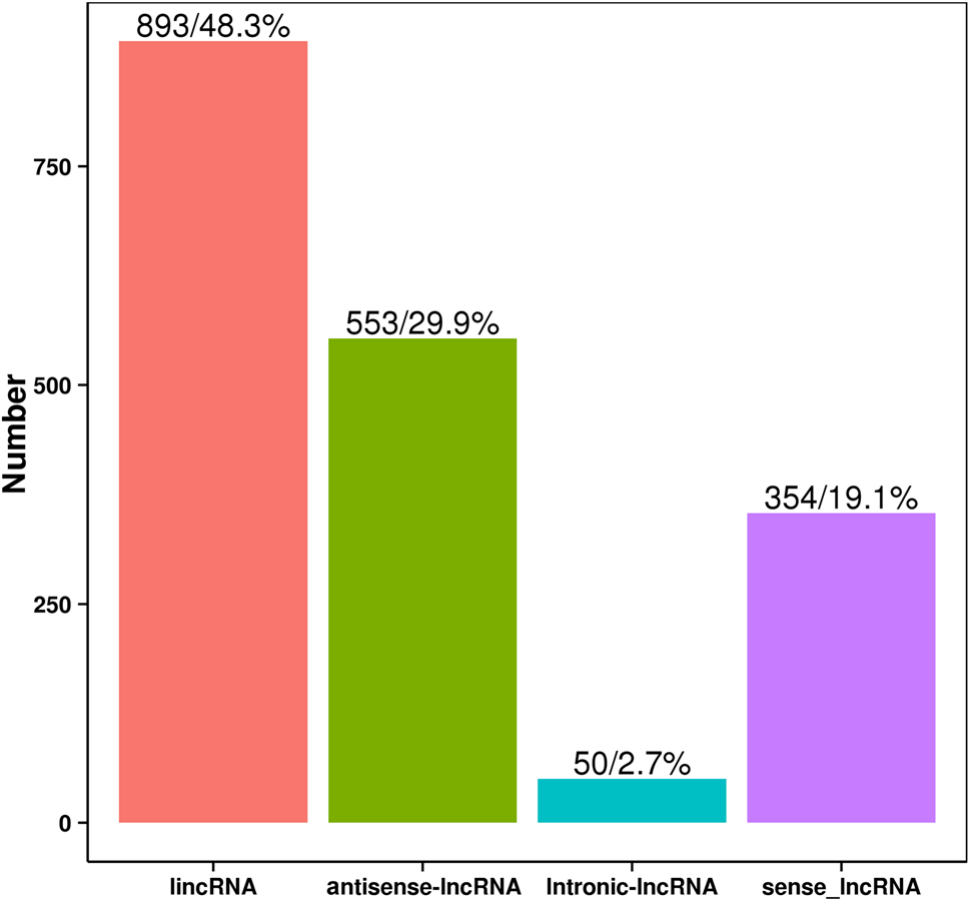
The types of lncRNA. The abscissa is the 4 types of lncRNA and the ordinate is the number of the corresponding lncRNA. LincRNA represents intergenic lncRNA.

Next, all transcripts with protein-coding potential were removed. The protein-coding potential of transcripts were predicted jointly by four analyses: CPC analysis (Coding Potential Calculator)^28^, CNCI analysis (Coding-Non-Coding Index)^29^, CPAT analysis (Coding Potential Assessment Tool)^30^ and Pfam protein domain analysis^31^. CPC is a protein coding potential calculation tool based on sequence alignment with known protein databases and the biological sequence characteristics of transcripts and it is noncoding RNA when Score < 0. CNCI analysis is a method to distinguish non-coding from coding transcripts by the traits of adjacent nucleotide triplets. It does not depend on known annotation files and can effectively predict incomplete transcripts and antisense transcripts, and transcript is noncoding RNA when score < 0. CPAT analysis is a method to judge transcript encoding ability by constructing logistic regression model, calculating coding probability based on ORF length and ORF coverage. When coding probability < 0.38, it is noncoding RNA. The Pfam database is the most comprehensive classification system for protein domain annotations. The transcripts with a high similarity with known protein domain were defined as transcripts with coding ability (E-value < 0.001). Thus, four computational approaches (CPC/CNCI/Pfam/CPAT) were combined to sort lncRNA candidates from putative protein-coding RNAs in the group of unknown transcripts. The lncRNAs candidates identified using the four methods were counted statistically and plotted in a Venn diagram, and the intersection of the four sets of lncRNAs were accepted as putative lncRNAs (Fig. 1). After potential coding ability screening, lncRNA candidates with potential coding ability were removed, and a total of 1,850 lncRNAs were the newly predicted lncRNAs. Four types of lncRNAs were obtained: long intergenic lncRNAs (lincRNAs), antisense lncRNAs, intronic lncRNAs and sense lncRNAs. The results indicated that there were 893 lincRNAs (48.3%), 553 antisense-lncRNAs (29.9%), 50 intronic-lncRNAs (2.7%), and 354 sense-lncRNAs (19.1%) (Fig. 2).

The lengths of lncRNAs ranged from 202 to 10,587 bp, with the vast majority (84%) having lengths shorter than 2000 bp (Supplementary Table S1), which is similar to that reported for potato (90%)^32^. The average length of Jatropha lncRNAs was 1,238 bp, which is significantly higher than that reported for potato (895), rice (800 bp) and chickpeas (614 bp)^32–34^. More than half (51%) of mRNAs have lengths longer than 2000 bp, indicating that the average length of lncRNAs was lower than that of mRNAs (Supplementary Figure S1). In this study, 1,386 lncRNAs had two exons; 322, three exons; 88, four exons; and 54, between five and eleven exons. On average, the exon numbers associated with *Jatropha* lncRNAs were lower than those associated with mRNAs (Supplementary Figure S1). These features of lncRNAs could also be found in other plants^32^.

### Quantitative analysis of lncRNAs

**Figure 3:**
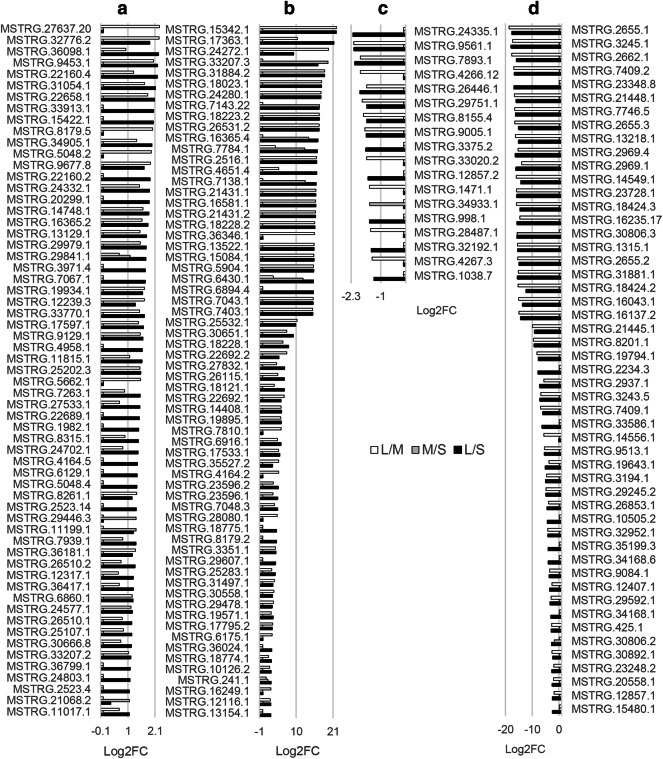
The differentially expressed lncRNAs during seed development of *J. curcas*. Differential expression analyses of lncRNAs were performed based on FPKMs of lncRNAs in each sample, which were calculated using the length of lncRNAs (kilo-base) and mapped fragments (million). The variation of lncRNA levels in three different developmental stages, i.e., small (S), middle (M) and large (L) seeds were shown as the base-2 logarithm of ratios (log_2_ fold change) between different developmental stages (L/M, M/S and L/S). The lncRNAs shown were differentially expressed lncRNAs because their expression levels between any two of the three different developmental stages changed significantly (|log_2_ fold change|> 1 and *p* < 0.01).

Plant lncRNAs play important functional roles in the regulation of plant growth and development. To gain insight into the roles of lncRNAs in *Jatropha*, the expression levels and patterns of lncRNAs in seed were determined at three different developmental stages. The lncRNA expression levels were presented as FPKM values which were comparable between different samples. Among the 1,850 lncRNAs identified in this work, about ten percent (196 lncRNAs) were differentially expressed lncRNAs (fold change > 2, and p < 0.01) (Supplementary Table S2). To compare the differential expression of lncRNAs between different development stages, the differential expression was presented as the base-2 logarithm of fold change of expression levels between small, middle and large stages (Fig. 3). In the 196 differentially expressed lncRNAs, 125 lncRNAs were up-regulated (Fig. 3a,b) whereas 69 lncRNAs were down-regulated (Fig. 3c,d), and the variation was continuous from small through large stages without fluctuation (Fig. 3). Only two lncRNAs changed without such rules. MSTRG.7207.40 lncRNA could be detected only in the middle stage of seed developing. On the contrary, MSTRG.20400.1 could NOT be detected in the middle stage of seed developing. Among the 125 up-regulated lncRNAs, about a half (61) lncRNAs were up-regulated less than five folds (Fig. 3a) while about a half (64) lncRNAs were up-regulated more than five folds (Fig. 3b); however, about 26% (18) lncRNAs were down-regulated less than five folds (Fig. 3c) while about 74% (51) lncRNAs were down-regulated more than five folds (Fig. 3d).

Interestingly, most differentially expressed lncRNAs (135 lncRNAs) were observed during seed development from middle to large ones, and only 16 lncRNAs (Supplementary Table S2) were observed differentially expressed when small seeds developed to middle seeds. Reverse transcription (RT) quantitative real-time PCR (qPCR) of 22 differentially expressed lncRNAs was then performed for the validation of the sequencing results. The RT-qPCR results of 13 down-regulated lncRNAs, 5 up-regulated lncRNAs, and 4 lncRNAs detected only in large seeds (MSTRG.25532.1, MSTRG.18228.2, MSTRG.17363.1 and MSTRG.4651.4) agreed well with the lncRNA expression profile displayed by the high-throughput sequencing data (Supplementary Figure S2). The differentially expressed lncRNAs were outlined in a heatmap (Supplementary Figure S3). Among three biological replicates, the overviews of the 196 differentially expressed lncRNAs were similar, and three clades were obtained from the samples of three different developmental stages accordingly, suggesting that the biological replicates were reliable, and three developmental stages could be used for differentially expressed lncRNA analysis (Supplementary Figure S3). A similar profile was observed between small seeds and middle ones, whereas a huge difference was observed in large seeds when compared with the small and middle seeds. This might be caused by similar metabolism happened in the small and middle seeds, where cell division, tissue differentiation and rapid growth were in progress, and drastic changes took place during the maturation process when dry matter accumulation started^27,35^. These results suggested that gene regulation of by lncRNAs was more active at the late seed developmental stages, which is much similar to the miRNA profile in the developing seeds of *Jatropha*^27^.

### Prediction and annotation of lncRNA targets

**Figure 4:**
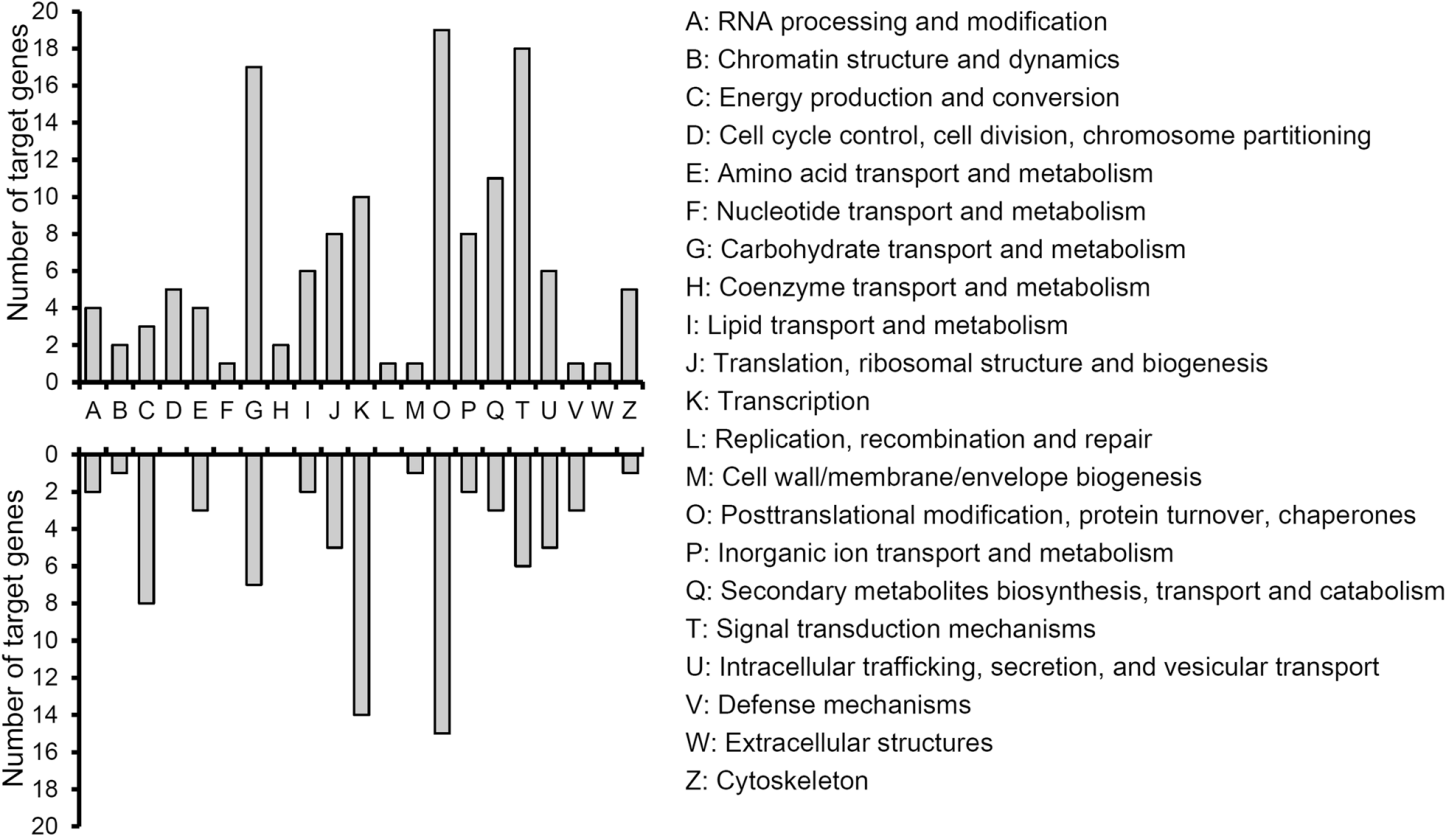
The function category of KOG annotation for the targets of differentially expressed lncRNAs during seed development of *J. curcas*. X-axis represents KOG category. Y-axis indicates the number of lncRNA target genes. Gene function was annotated on the basis of KOG/COG (Clusters of Orthologous Groups of proteins). The genes with unknown function were not counted in. Upper panel, target genes of up-regulated lncRNAs; lower panel, target genes of down-regulated lncRNAs.

Based on the mode of interaction between lncRNA and its target gene, we adopted two prediction methods. First, lncRNA regulates the expression of its adjacent genes, and predicts that the adjacent genes within the range of 10 kb of lncRNA are its target genes according to the location relationship between lncRNA and gene. Second, lncRNA might play a role on RNA due to complementary base pairing, and therefore LncTar was used to predict lncRNA target gene^36^. The target genes were found for a total of 1563 lncRNAs based on the two prediction methods (Supplementary Table S3). The targets of differentially expressed lncRNAs were annotated on the basis of KOG/COG. According to their physiological and biochemical functions, except for those genes with unknown function, the target genes are mainly involved in three aspects of plant physiological process: gene expression regulation, metabolism, and cell growth and development. Interestingly, among those 211 target genes with explicit KOG function, nearly half of the target genes (45%) are related to gene expression regulation, including signal transduction mechanisms (T, 24), transcription (K, 24), translation (J, 13), and posttranslational modification (O, 34). The second largest group of targets are involved in metabolism (35%), including carbohydrate transport and metabolism (G, 24), lipid transport and metabolism (I, 8), amino acid transport and metabolism (E, 7), inorganic ion transport and metabolism (P, 10), secondary metabolites biosynthesis, transport and catabolism (Q, 14), and energy production and conversion (C, 11). This is consistent with previous observations in other oil crops, such as castor bean^21^ and *Brassica napus*^19^, in which lncRNA is an important regulator involved in carbon metabolism and lipid metabolism. The third group of target genes function in cell growth and development (11%), such as intracellular trafficking, secretion, and vesicular transport (U, 11), cytoskeleton (Z, 6), cell wall biogenesis (M, 2), and cell cycle control (D, 5) (Fig. 4). These results indicated that the differentially expressed lncRNAs might play an important role in the seed developing and substance accumulation by regulating many target genes involved in gene expression regulation, metabolism, and cell growth.

### The relationship of lncRNAs and miRNAs in *Jatropha*

In plants, a group of endogenous small non-coding RNAs named microRNAs (or miRNAs) target mRNAs for cleavage or translational repression. miRNAs are initially transcribed as long polyadenylated transcripts called pri-miRNAs which are processed into shorter miRNA precursors (pre-miRNAs), and these pre-miRNAs are further processed into 18–24 nucleotide (nt) mature miRNAs^37^. A total of 10 lncRNAs was found to be miRNA precursors by mapping miRNAs which were identified from our previously sequenced small RNA libraries^27^ to the 1,850 lncRNAs determined in this work, suggesting that some lncRNAs encode miRNAs in *Jatropha* (Supplementary Table S4). These miRNAs might be important regulators for seed development. For example, a target of miR168 (coded by lncRNA MSTRG.30828.3) was AGO1, which was essential for miRNA maturation^38^, and the interaction between miR168 and AGO1 maintained proper embryo development^39^. In addition, miR168 was shown to be involved directly in lipid biosynthesis in the developing seeds of another woody oil plant (sea buckthorn)^40^. miR396a (from lncRNA MSTRG.24217.2) targets growth-regulating factor (GRF) which is shown to control plant seed development^41^.

Furthermore, 26 lncRNAs were predicted to be the targets of *Jatropha* miRNAs (Supplementary Table S5). These lncRNAs showing complementarity to miRNAs, might act as decoys, competing with mRNAs for binding to miRNAs to regulate genes involved in seed development. Target mimicking of miRNA is one of the most important mechanisms of lncRNAs regulating the plant development^11,42^. In other plant species, such as chickpea, Arabidopsis, citrus, rice, canola and maize, it was shown that lncRNAs also could act as miRNA targets, miRNA precursors or endogenous target mimics^34,42–46^. To sum up, the interaction between lncRNAs and miRNAs could be an important posttranscriptional regulatory mechanism for gene regulation in the developing seeds of *Jatropha*.

### The key lncRNAs involved in seed development

**Figure 5:**
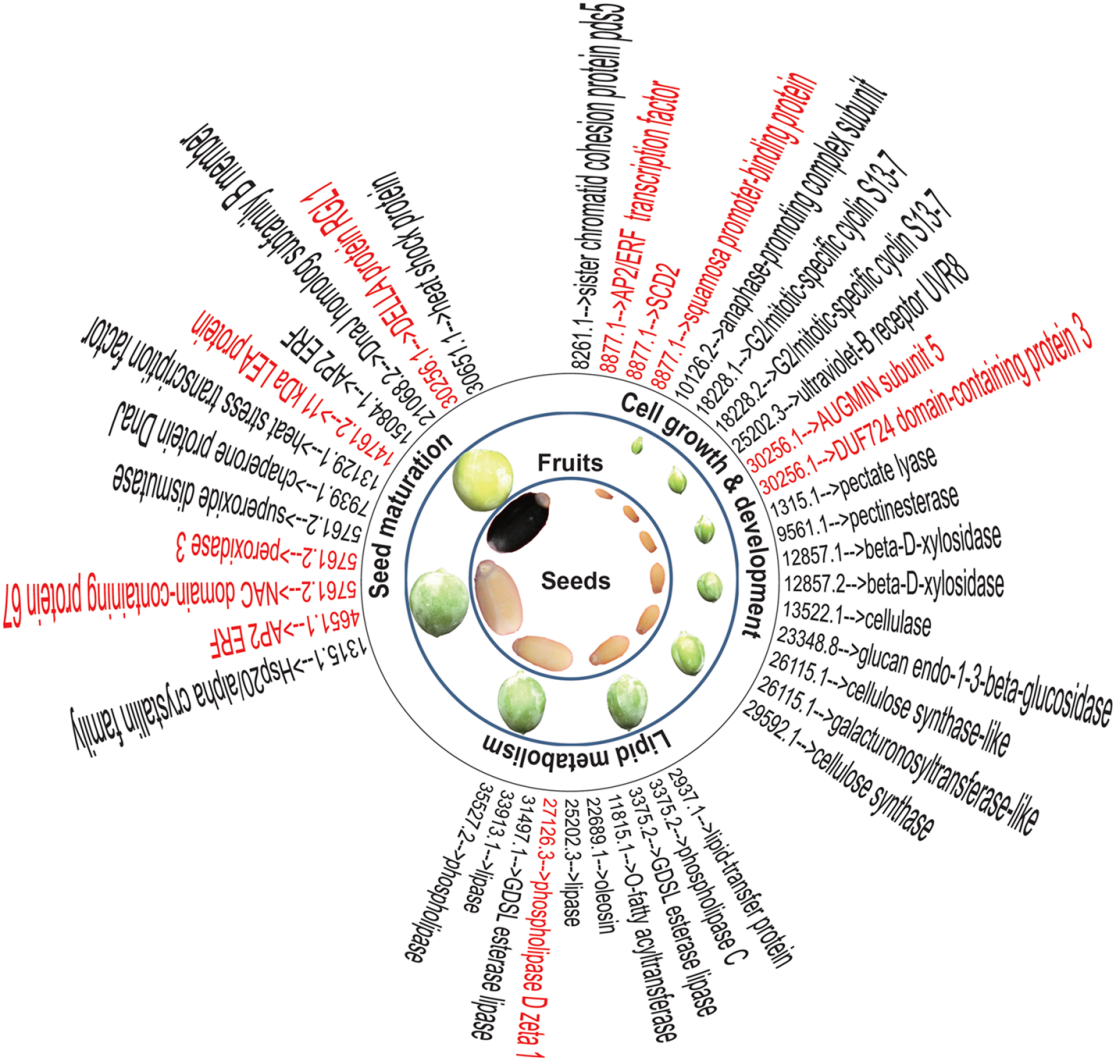
Key lncRNAs in the context of cell growth and development, lipid metabolism, and seed maturation of *Jatropha* developing seeds. Numbers before the arrows are lncRNA ID, and the targets of lncRNA are shown after the arrows. The lncRNAs might act as endogenous target mimics of miRNA, and the miRNA targets, are shown in red (For detail, see Supplementary Table S7).

To be more specific about the roles of differentially expressed lncRNAs in seeds, lncRNAs were refined in the context of possible functions in the three aspects of oil seed development, i.e., cell growth and development, lipid metabolism, and seed maturation, and eventually 31 key lncRNAs were obtained (Fig. 5 and Supplementary Table S7).

Seven lncRNAs (MSTRG.8261.1, MSTRG.8877.1, MSTRG.10126.2, MSTRG.18228.1, MSTRG.18228.2, MSTRG.25202.3, and MSTRG.30256.1) were implicated in cell division according to the annotated function of their targets in the UniProt databases. Some targets participate in several stage of cell cycle, such as G2/mitotic-specific cyclin S13-7, anaphase-promoting complex subunit, and sister chromatid cohesion protein pds5. Likewise, some targets are required for cell division, including AUGMIN subunit 5 implicated in spindle assembly, SCD2 in cytokinesis, and DUF724 domain-containing protein 3 in the polar growth of plant cells^47^. AP2/ERF transcription factors and squamosa promoter-binding protein are both shown to control plant seed development^48–50^. UVR8 (target of lncRNA MSTRG.25202.3) is required for normal progression of endocycle, which is endoreduplication or DNA replication without mitosis, leading to the formation of nuclear type endosperm. This agrees well with the fact that endosperm of *Jatropha* is nuclear type in the early stage of seed development^51^. At least 8 lncRNAs (MSTRG.1315.1, MSTRG.9561.1, MSTRG.12857.1, MSTRG.12857.2, MSTRG.13522.1, MSTRG.23348.8, MSTRG.26115.1, MSTRG.29592.1) were associated with the formation of cell wall and seed coat development, as most of their targets are related to the synthesis of cellulose and pectate which are required for cell growth. In addition, previous work showed that beta-D-xylosidase (target of MSTRG.12857.1) play important roles in seed coat development^52^.

Seed oil is always the focus of *J, curcas*, a promising biodiesel plant. Interestingly, several important proteins involved in lipid metabolism were found to be the target of 9 differentially expressed lncRNAs, including lipase, lipid-transfer protein, O-fatty acyltransferase, oleosin, and non-specific lipid-transfer protein (Fig. 5). In oil seeds, oil is stored in oil body surrounded by a half-unit phospholipid membrane containing oleosin, and oleosin is the major protein constituent of *Jatropha* oil body^53^. It agrees with previous reports that the oleosin is the target of lncRNA in the developing seed of *Brassica napus* and pigeonpea^19,22^. The non-specific lipid-transfer proteins facilitate the transfer of fatty acids and phospholipids between membranes. Mean while, phospholipase D zeta regulated by MSTRG.27126.3 through miR827 enhance diacylglycerol flux into triacylglycerol^54^. These results suggested that lncRNAs act pivotal part in the oil accumulation of *Jatropha* seeds.

Many target genes regulated by lncRNAs have critical roles in seed maturation process during which seeds suffering dehydration stress. For example, heat shock protein (including DnaJ), AP2 ERF, and heat stress transcription factor may be involved in the regulation of gene expression by stresses. Peroxidase and superoxide dismutase are known antioxidant proteins, which may protect seeds from injury of free radical. NAC domain-containing protein 67 and late embryogenesis abundant protein (LEA) may play an essential role in seed survival during seed dehydration stress by maintaining cellular stability^55^. This is in agreement with a previous report that LEA protein was a lncRNA target in the developing seeds of pigeonpea^22^. DELLA protein my contribute to the regulation of seed dormancy process, which is also an important stage of seed maturation. Collectively, these observations suggest that lncRNAs play a pivitol part in the regulation of desiccation tolerance and antioxidant system during *Jatropha* seed maturation.

## Conclusions

In summary, we screened and identified 1,850 lncRNAs followed by examining their expression patterns at three different developmental stages of *J. curcas* seeds. The possible roles were investigated for lncRNAs, including target gene regulator, miRNA precursor and miRNA target. Functional analysis of the target genes of differentially expressed lncRNAs showed that lncRNAs play important parts in multiple biological processes, such as posttranslational modification, carbohydrate metabolism and signal transduction. Further analysis showed that 31 key lncRNAs could function in the seed developing process in the context of cell growth and development, lipid metabolism, and seed maturation. It indicated that the up- or down-regulation of lncRNAs is an important mode to regulate the process of seed developing. This study reveals expression profiles of lncRNAs in seed developing, providing important data for further investigation on the mechanisms of molecular regulation of seed development.

## Materials and methods

### Seed collection and RNA isolation

It was performed by the same method as reported previously^27^. Fruits from *J. curcas* were collected randomly from 6 plants at 5–10 (small seeds), 12–20 (middle seeds) and 25–35 (large seeds) days after flower opening (DAF). Small, middle and large seeds represent three typical stages of seed developing, i.e., young, intermediate and mature. Seeds at each stage were collected and frozen immediately in liquid nitrogen and stored at -80 °C. Total RNA was isolated from a pool of seeds from each stage with Trizol (Invitrogen, CA, USA) according to the manufacturer’s protocol. RNAs were prepared from three independent biological replicates.

### Preparation for lncRNA library

RNA libraries were constructed following methods described in Zuo et al^56^. In brief, a total of 1.5 μg RNA per sample was used as input material for rRNA removal using the Ribo-Zero rRNA Removal Kit (Epicentre, Madison, WI, USA). Sequencing libraries were prepared by using NEBNext^R^ Ultra™ Directional RNA Library Prep Kit for Illumina^R^ (NEB, USA) following manufacturer’s protocol. Fragmentation was carried out in NEB Next First Strand Synthesis Reaction Buffer (5 ×) under elevated temperature. First strand cDNA was synthesized using reverse transcriptase and random hexamer primer. Second-strand cDNA synthesis was performed subsequently using RNase H and DNA Polymerase I. NEB Next Adaptor with hairpin loop structure were ligated to prepare for hybridization after adenylation of 3′ ends of DNA fragments. The library fragments were purified with AMPure XP Beads (Beckman Coulter, Beverly, USA) to select insert fragments of preferentially 150 ~ 200 bp in length. Then PCR was performed with universal PCR primers and Phusion High-Fidelity DNA polymerase. Finally, PCR products were purified (AMPure XP system), followed by library quality assessment on the Agilent Bioanalyzer 2,100. After sequencing with Illumina HiSeq2500 platform (San Diego, CA, USA), the paired-end reads were generated. Three independent RNA libraries were constructed from each of the three stages of the developing seeds resulting in nine lncRNA libraries.

### Sequence data analysis

Sequence data analysis was performed according to procedures previously reported^56^. In brief, from raw reads in fastq format, reads containing adapter, reads containing ploy-N and low-quality reads were removed to obtain clean data (clean reads) with high quality. Clean reads of each sample were mapped to the *J. curcas* genome (JatCur_1.0, https://www.ncbi.nlm.nih.gov/assembly/GCF_000696525.1/) using HISAT2^57^, and the mapped reads was subjected to further de novo assembly and quantification by StringTie^58^. The gffcompare program was used to annotate the assembled transcripts. Putative lncRNAs were screened from the unknown transcripts. To overcome transcriptional noise, two or more exons, length greater than 200 bp, and abundance greater than 0.5 FPKM in at least one of the samples were selected as lncRNA candidates. These candidates were further screened using four computational approaches (CPC/CNCI/Pfam/CPAT) which can distinguish the non-coding genes from the protein-coding genes. The different types of lncRNAs include lincRNA, intronic lncRNA, anti-sense lncRNA, and sense lncRNA were selected using cuffcompare.

### Quantification of gene expression levels and differential expression analysis

StringTie (1.3.1) was used to calculate FPKMs of lncRNAs in each sample. FPKM means fragments per kilo-base of transcript per million fragments mapped, calculated based on the length of the transcript length (kilo-base) and mapped fragments (million). Differential expression analysis was performed using the DESeq R package (1.10.1) following the procedures described previously^27^. Benjamini and Hochberg’s approach was adopted to adjust the P values to controlling the false discovery rate. Genes with absolute value of log_2_(Fold change) > 1 and adjusted P value < 0.01 were assigned as differentially expressed genes.

### Reverse transcription quantitative real-time PCR (RT-qPCR)

RT-qPCR was performed following previously established procedures^27^. In brief, total RNA was treated with DNase I followed by reverse transcription using random primers and SuperScript™ III reverse transcriptase (Invitrogen) according to the manufacturer’s instructions. Two-step Real-time PCR was performed in ABI StepOne (USA) following a standard SYBR Premix Ex Taq II (TaKaRa) protocol: 95 °C for 5 min, and 40 cycles of 95 °C for 10 s and 60 °C for 30 s. The differences in gene expression were calculated using the 2^−ΔΔCt^ analysis method^59^ using the actin (GenBank: HM044307.1) as internal reference gene and the young seeds as control for gene expression normalization. Each reaction was performed in triplicate. All primers used in RT-qPCR experiments were listed in Supplementary Table S6.

### Prediction and annotation of lncRNA targets

The adjacent genes within the range of 10 kb of lncRNA are predicted to be its target genes according to the location relationship between lncRNAs and genes. LncTar was used to predict lncRNA target gene based on complementary base pairing^36^. LncTar uses complementary sequences between lncRNA and RNA to calculate the free energy and standardized free energy. When the free energy of pairing sites between RNA and lncRNA was below the threshold of standardized free energy (< − 0.1), the RNA was considered to be a target of the lncRNA. Gene function was annotated based on KOG/COG database (Clusters of Orthologous Groups of proteins; https://www.ncbi.nlm.nih.gov/KOG).

### Prediction lncRNAs targeted by miRNAs

The miRNA target prediction was performed by aligning the mature miRNA sequences against *J. curcas* lncRNA sequences using psRNAtarget with default parameters except for a strict Expectation value 3^60^.

## Supplementary information

Supplementary Figures.

Supplementary Table S1.

Supplementary Table S2.

Supplementary Table S3.

Supplementary Table S4.

Supplementary Table S5.

Supplementary Table S6.

Supplementary Table S7.

## Data Availability

All sequencing data were deposited in the NCBI Sequence Read Archive under ID SRR7640476 (small seeds), SRR7640477 (middle seeds) and SRR7640478 (large seeds). All data generated or analyzed during this study are included in this published article and its Supplementary Information files.
