# Supplementary material for: Identification and characterization of long non-coding RNA (lncRNA) in the developing seeds of Jatropha curcas
Source: Sci Rep. 2020 Jun 25;10:10395. doi: 10.1038/s41598-020-67410-x (PMC7316758; doi:10.1038/s41598-020-67410-x)
Supplement: Supplementary file 1 — Supplementary Figures. [file 41598_2020_67410_MOESM1_ESM.docx]

Identification and characterization of Long Non-Coding RNA (lncRNA) in the developing seeds of *Jatropha curcas*

Xihuan Yan^1,2^, Lanqing Ma^1,2^*, MingFeng Yang^2^*

1. Beijing Advanced Innovation Center for Tree Breeding by Molecular Design, Beijing University of Agriculture, Beijing 102206, P. R. China

2. Key Laboratory for Northern Urban Agriculture of Ministry of Agriculture and Rural Affairs, Beijing University of Agriculture, Beijing 102206, P. R. China

* [lqma@bua.edu.cn](mailto:lqma@bua.edu.cn) or [mfyang@bua.edu.cn](mailto:mfyang@bua.edu.cn)

Xihuan YAN

[1300869026@qq.com](mailto:1300869026@qq.com)

Lanqing MA

[lqma@bua.edu.cn](mailto:lqma@bua.edu.cn)

Mingfeng YANG

[mfyang@bua.edu.cn](mailto:mfyang@bua.edu.cn)

^*^ Corresponding author: Mingfeng YANG

Key Laboratory for Northern Urban Agriculture of Ministry of Agriculture and Rural Affairs, Beijing University of Agriculture, Beijing 102206, P. R. China
E-mail: lqma@bua.edu.cn
or mfyang@bua.edu.cn

Supplementary Figure S1.

Figure S1. The difference between lncRNA and mRNA in the developing seeds of *Jatropha*. (a) The length distribution of lncRNA/mRNA is shown in the statistical map. The abscissa is the length, and the ordinate is the number of lncRNA/mRNA in the length distribution range. (b) The number of exons corresponding to lncRNA/mRNA is shown in the statistical chart. The abscissa is the number of exons and the ordinate is the number of lncRNA/mRNAs in the exon distribution range.

Supplementary Figure S2

Figure S2. RT-qPCR validation for the differentially expressed lncRNAs. The relative expression level of down-regulated lncRNAs (a) and up-regulated lncRNAs (b) at young (small), intermediate (middle), and mature (large) stage of seed development are shown. The lncRNAs whose expression levels in large seeds changed significantly (p <0.05, t-test) compared with small seeds were assigned as differentially expressed lncRNAs (indicated by *). The LncRNAs whose expression levels in large seeds changed significantly (p <0.05, t-test) compared with middle seeds were assigned as differentially expressed lncRNAs (indicated by #). MSTRG.25532.1, MSTRG.18228.2, MSTRG.17363.1 and MSTRG.4651.4 could be detected only in large seeds. Values are the mean ± s.d. of three biological replicates.

Supplementary Figure S3


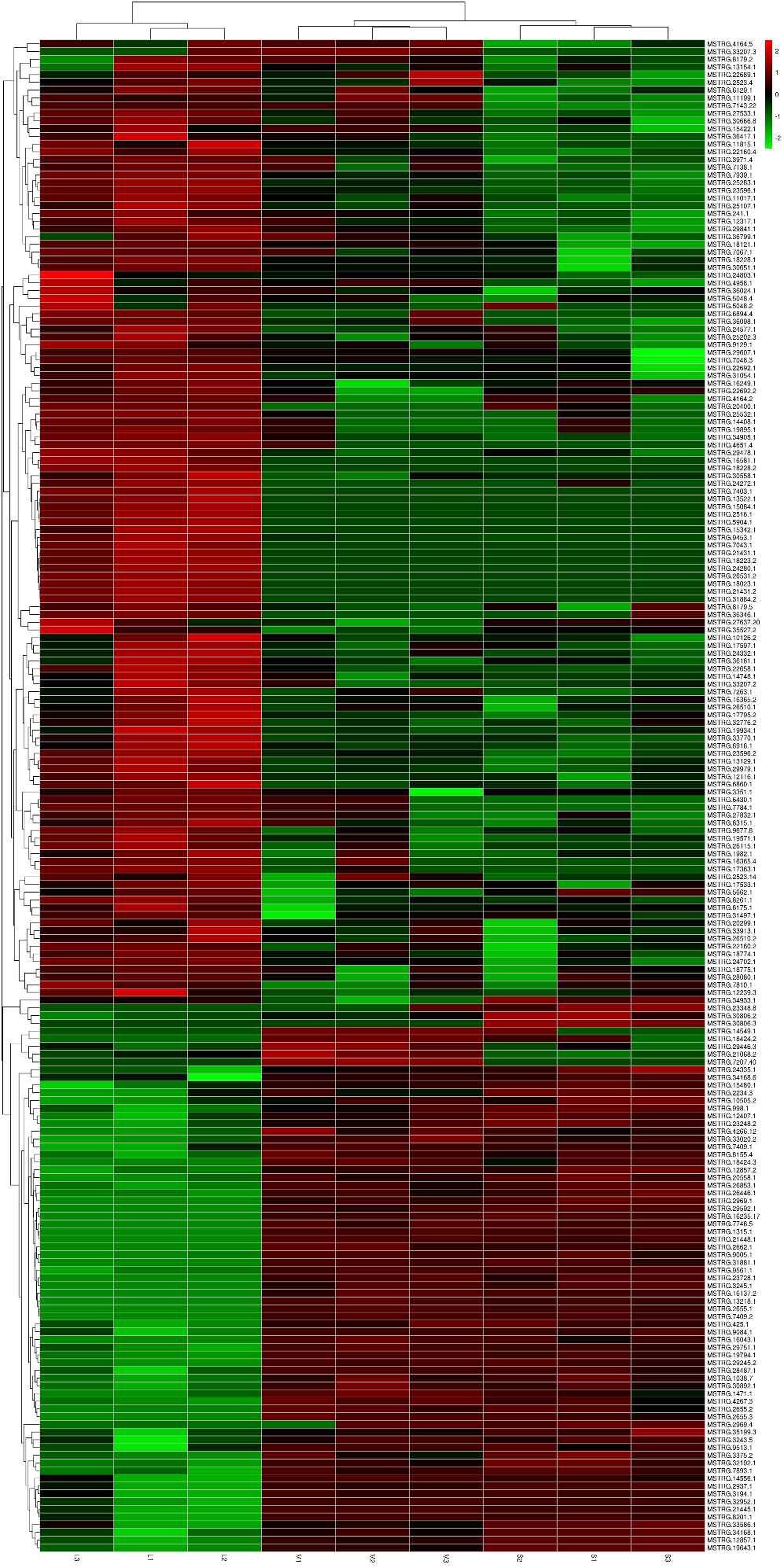


Figure S3. The global expression of the 196 differentially expressed lncRNAs in three developmental stages (small, middle and large) of *J. curcas* seeds. Three independent biological replicates of developing seeds at each stage (small: S1, S2 and S3; middle: M1, M2, and M3; large: L1, L2, and L3) were shown. The colors indicate relative expression levels of lncRNAs, where high levels are indicated by red, and low levels are green.
